# OXA-244-Producing ST131 *Escherichia coli* From Surface and Groundwaters of Pavia Urban Area (Po Plain, Northern Italy)

**DOI:** 10.3389/fmicb.2022.920319

**Published:** 2022-06-09

**Authors:** Aseel AbuAlshaar, Aurora Piazza, Alessandra Mercato, Federica Marchesini, Vittoria Mattioni Marchetti, Ibrahim Bitar, Jaroslav Hrabak, Melissa Spalla, Giorgio Pilla, Renato Sconfietti, Roberta Migliavacca

**Affiliations:** ^1^Unit of Microbiology and Clinical Microbiology, Department of Clinical-Surgical, Diagnostic and Pediatric Sciences, University of Pavia, Pavia, Italy; ^2^Department of Microbiology, Faculty of Medicine, University Hospital in Pilsen, Charles University, Pilsen, Czechia; ^3^Department of Earth and Environment Sciences, University of Pavia, Pavia, Italy

**Keywords:** ST131 *Escherichia coli*, OXA-244, ST258 *Klebsiella pneumoniae*, ESβLs, *Enterobacterales*, carbapenemases, river and ground waters, Italy

## Abstract

The study aimed to investigate (i) the occurrence of third-generation cephalosporins and/or carbapenems non-sensitive *Enterobacterales* in Pavia surface and groundwaters, (ii) their resistance determinants, and (iii) the clonal features of the most relevant strains. During May 13 and 14, 2019, *n* = 18 water samples from *n* = 12 sampling sites in the urban/peri-urban area of Pavia (Po Plain, Northern Italy) have been evaluated. At first, hydrochemical analysis and bacterial plate counts were carried out on all the water samples. One milliliter of each water sample was then screened on both MacConkey agar (MC) added with cefotaxime (1 mg/L; 2 mg/L) and MC plus meropenem (0.25 mg/L; 4 mg/L). Species identification and antimicrobial susceptibilities were assessed by MicroScan autoSCAN-4. Double Disk Synergy (DD) test, CT103XL microarray, *acc(6‘)-Ib-cr, qnr*S, *bla*CTX-M-/MOX-/VEB-/OXA-type genes targeted PCR and sequencing, Pulsed-Field Gel Electrophoresis (PFGE), MultiLocus Sequence Typing (MLST), and Whole-Genome Sequencing on selected strains were performed. A total of *n* = 30 isolates grown on β-lactams enriched MC: *Escherichia coli* (*n* = 21; 70%), *Klebsiella* spp. (*n* = 5; 16.6%), *Citrobacter freundii* (*n* = 2; 6.7%), and *Kluyvera intermedia* (*n* = 2; 6.7%). All *E. coli* and *K. pneumoniae* were ESβL-producers by DD. The 66.6, 38.0, and 19.0% of *E. coli* were ciprofloxacin/levofloxacin, trimethoprim-sulfamethoxazole, and gentamicin resistant (EUCAST 2019 breakpoints), respectively. A *bla*CTX-M-type determinant was identified in *E. coli* (*n* = 20/21; 95.2%) and *K. pneumoniae* (*n* = 2/3; 66.7%). The remaining *E. coli* was *bla*VEB-1 and *bla*MOX-2 genes positive. The *aac*(6′*)-Ib-cr* determinant was found in *n* = 7 *E. coli* and *n* = 1 *K. pneumoniae*, while *qnrS* was found in *n* = 1 *E. coli* and *n* = 2 *K. pneumoniae*. PFGE showed clonal heterogeneity among ESβL-*E. coli*. Two out of four *E. coli* detected as *bla*OXA-244-positive, belonged to the pandemic ST131. One XDR *K. pneumoniae* from a stream sample, detected as *bla*KPC-2 positive, resulted of ST258. The epidemiological impact of *bla*OXA-244 ST131 *E. coli* and *bla*KPC-2 ST258 *K. pneumoniae* presence in surface waters of an urban area in Northern Italy must not be underestimated.

## Introduction

The increasing rise of antimicrobial resistance (AMR) is one of the greatest threats to human health in the 21st century. An increasing body of research identifies the environment as not only a recipient of drug-resistant bacteria but also as a reservoir and source of resistance genes (Nappier et al., 2020). The presence of antimicrobial-resistant bacteria and related antimicrobial-resistant genes (ARGs) in the environment is now well-recognized for its role in the spread of AMR. Numerous ARGs associated with human diseases have an environmental origin. Several studies have focused on monitoring and treatment of ARGs in different environmental matrices. ARGs in wastewater treatment plants (WWTPs) are being studied from multiple perspectives due to the reuse of wastewater for agricultural purposes—often without extensive treatment (Waseem et al., 2017). The routes by which humans may come in contact with these bacteria include the consumption of crops grown by contaminated sludge used as fertilizer and/or drinking of water drawn from the contaminated ground or surface water (Caltagirone et al., 2017). The World Health Organization (WHO) and European Centre for Disease Prevention and Control (ECDC) recognized AMR as one of the most important public health problems of the 21st century, which needs to be immediately resolved. The fact that it was the main subject of the G20 summit (September 2016, Hangzhou, China), as well as the General Assembly of the United Nations (September 2016, New York, USA), reflects the seriousness of the situation. Antibiotics were also used in promoting growth and preventing disease in livestock. The consequence of their high consumption, incorrect medication disposal methods, or excretion by humans and animals is the getting through of antibiotics, their metabolites, and transformation products into hospital and municipal sewage. In the environment, antibiotics are not only chemical pollutants that can exert toxic effects but also above all to be able to cause selection pressure. This phenomenon consists of the elimination of microorganisms (sensitive to antibiotics) and the survival of resistant cells, the characteristics of which allow them to overcome the adverse effects of antibiotics. Wastewaters after the treatment process are discharged to the receiving environments, such as surface waters (rivers, lakes, seas, or oceans), or into agricultural soils and crops as the result of irrigation with reclaimed wastewater, where the resistance may be transmitted between bacteria. For this reason, the WWTPs are reservoirs of resistance (Pazda et al., 2019). There is accumulating data revealing an inter-exchange of these genes between wildlife, livestock, and humans. Carbapenem-resistant *Enterobacterales* (CRE) isolates have been found in samples of different origins in many countries in recent years, including wastewaters, vegetables, animals, drinking water, wells, and river water. Rivers are remarkable hotspots of AMR, especially when exposed to human activities (Teixeira et al., 2020).

Aims of the study were (i) to investigate the occurrence of third-generation cephalosporins (3GCs) and carbapenems non-susceptible *Enterobacterales* in Pavia surface water compartment, (ii) to characterize the highest impact resistance determinants of the above isolates, and (iii) to assess the clonal features of the most relevant strains.

## Materials and Methods

### Study Area and Sampling Sites

Eighteen water samples were collected from the surface and groundwater of urban and peri-urban areas of Pavia (Northern Italy) in a 2-day sampling period, May 13 and 14, 2019.

**Figure 1:**
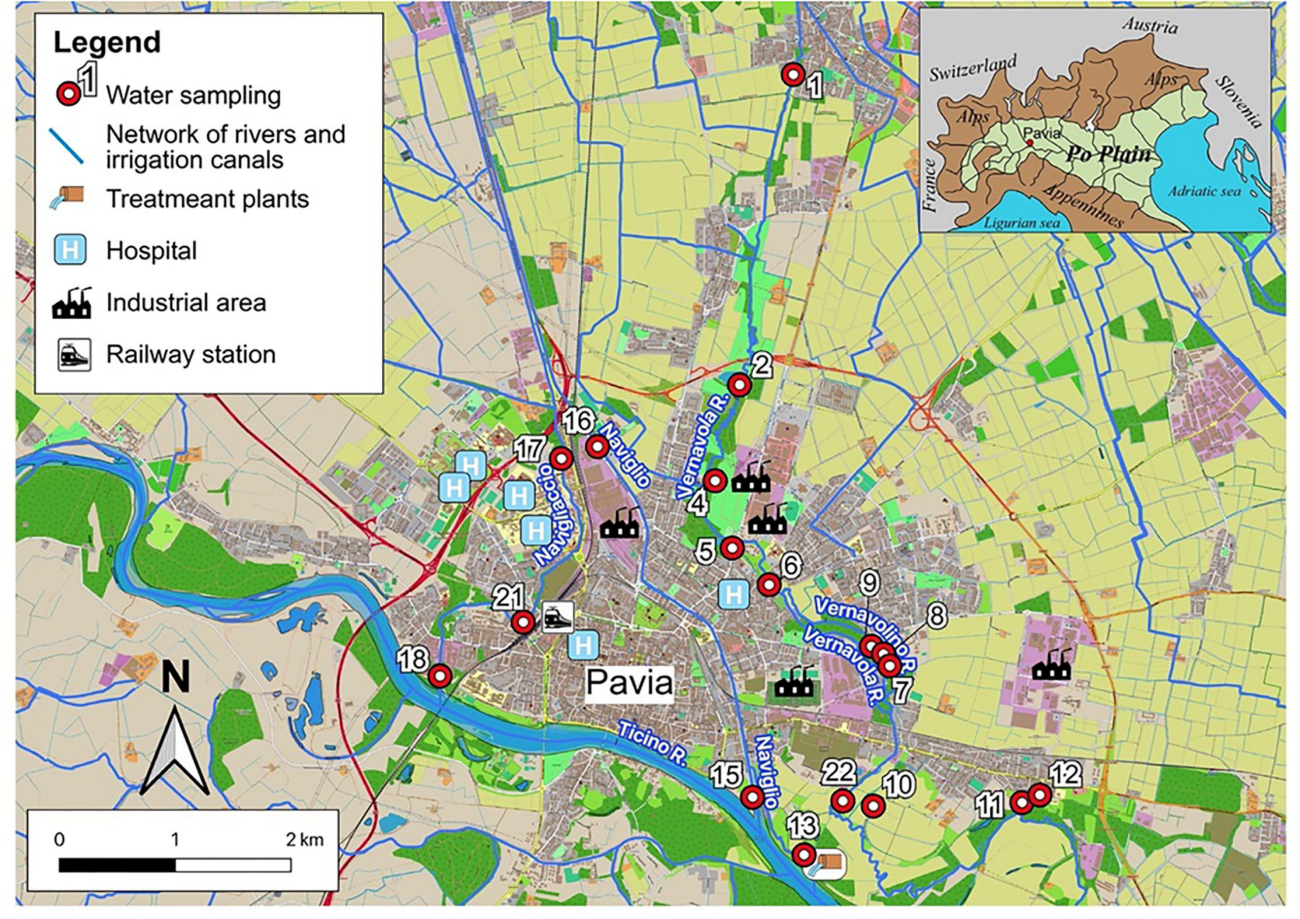
Map of the urban area of Pavia; the watercourses are indicated by names and the sampling sites by numbers.

**Figure 2:**
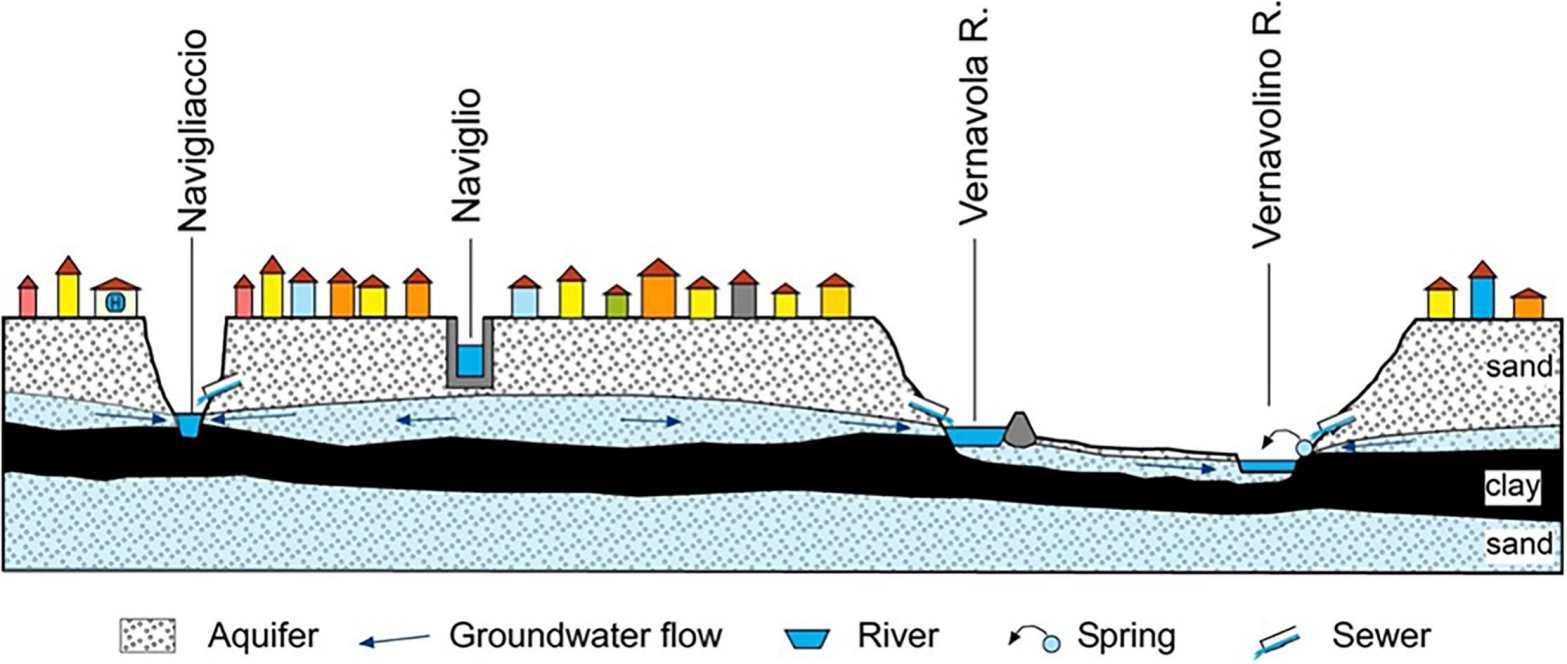
Schematic hydrogeological section through the city of Pavia.

Urban watercourses, both natural and of anthropogenic origin, were taken into consideration, intentionally excluding the Ticino river, as a very large hydrological basin, only marginally lapping the city. Each watercourse was sampled in at least two points, upstream (at the entrance) and downstream (at the exit) of the urban area. The sampling campaigns were carried out in the Roggia Vernavola valley (including the semi-natural Roggia Vernavola, once used for irrigation purposes, mainly; and the artificial Vernavolino canal), along the semi-natural Navigliaccio watercourse, and along the Naviglio Pavese canal of artificial origin (Figure 1). During their course in the Pavia urban area, the first three watercourses receive a significant groundwater supply from a perched aquifer located in the first meters of the underground (Figure 2). The Naviglio Pavese canal flows within a substantially impermeable riverbed and has no contact with underground waters (Figure 2). In addition to the watercourses, water bodies fed by the surface aquifer and small springs, mostly from fluvial terraces, were also sampled.

The watercourses sampled show the following features:

- the Roggia Vernavola originates just north of Pavia, in the municipality of San Genesio ed Uniti. In the first section, it runs through the homonymous urban park; after the above-mentioned park, it crosses Pavia city and flows into the Ticino river to the south-east of the city. It receives a few, but relatively important, direct sewage drains and several floods drains from the sewer collector. The Vernavola stream water was sampled in three points: sample 1, north of Pavia; sample 6, in the Pavia urban area; and sample 22, in the floodplain of the Ticino river, downstream of the urbanized area (Figure 1);
- the Vernavolino canal runs alongside the Roggia Vernavola in the urban area. During its course, it collects small sewage drains. The Vernavolino canal water was sampled in two sites: sample 7, in the urban area; and sample 10 along the Ticino river floodplain (Figure 1);
- the Naviglio Pavese canal flows from Milan with a north-south route; once used for commercial navigation, it is currently used for irrigation purposes. It flows into the eastern sector of Pavia city, about 2 km upstream from the mouth of the Roggia Vernavola. The Naviglio Pavese canal water was sampled in two sites: sample 16, at the entrance to the Pavia city; and sample 15, near the mouth of the Ticino river (Figure 1);
- the Navigliaccio canal, coming from Milan as the Naviglio Pavese, laps the city in its western portion and flows south into the Ticino river. Three sampling points were chosen along the Navigliaccio canal: sample 17 at the entrance to Pavia city; sample 21 in the urbanized area; and sample 18 near the mouth of the Ticino river (Figure 1).

The other samples were of various origins. We collected water from spring sources fed by a perched aquifer (samples 4 and 8), a quarry small lake fed by groundwater and probably sewage drains (samples 2 and 9), and the outflow of the treatment plants of Pavia city (sample 13).

In the eastern sector of the city, near the Ticino river escarpment, a spring, coming from the perched aquifer (sample 12), and an oxbow lake of an old riverbed of the Ticino river, fed by groundwater, were sampled (sample 11).

Finally, in the urban area of Pavia, a small artificial pond was sampled (sample 5), whose waters drain the surface aquifer.

### Chemical Analyses of Water Samples

The presence and abundance of the major ions were analyzed with a Dionex DX 120 chromatography, while volumetric analysis was used for the determination of alkalinity. All reported values have an ionic balance within 5%. A WTW LF597 conductivity meter for acquiring electrical conductivity and temperature data and a WTW pH 340/ion for acquiring pH data were used. For the analysis of the Chemicals Oxygen Demand (COD), the Spectroquant® analytical kits (Merck) and the NOVA 60 photometer (Hach) were used.

### Sample Filtration and Bacterial Counts

From each water sample two different volumes, 250 μl and 500 μl, were diluted in 1 ml of distilled water and filtered through 0.45 μm-pore size membrane filters. The filters were placed on Plate Count Agar (PCA), MacConkey Agar (MC), selective for non-wild type strains potential ESβLs producers, supplemented with either 1 μg/ml of Cefotaxime (CTX) or 2 μg/ml of CTX, and selective for non-wild type strains potential carbapenemase producers, supplemented with either 0.25 μg/ml of meropenem (MEM), or 4 μg/ml of MEM. The total bacterial count was estimated in triplicates after incubation of plates at 37°C for 24 h. The average of the counts was expressed in colony-forming units per ml (CFU/ml).

### Species Identification and Antimicrobial Susceptibility Testing

Species identification and susceptibility profiles were obtained using the semi-automated system MicroScan autoSCAN-4 (Beckman Coulter, Milan, Italy). Susceptibility results were interpreted according to the EUCAST 2019 guidelines (www.eucast.org). Colistin (CO) Minimum Inhibitory Concentration (MIC) values were confirmed using a UMIC broth microdilution kit (Biocentric, Bandol, France) for strains showing CO resistance with the antibiogram.

### Phenotypic and Molecular Investigation of ESβLs and Carbapenemases

The production of ESβLs was evaluated by the Double-Disc Synergy (DD) test with piperacillin-tazobactam, cefotaxime, cefepime, ceftazidime, and aztreonam disks on Mueller Hinton agar plates, according to EUCAST 2019 guidelines (www.eucast.org). The phenotypic detection of carbapenemases was performed using the KPC/MBL and OXA-48 Confirm kit (Rosco Diagnostica A/S, Taastrup, Denmark).

The genomic DNA of the strains was extracted using the Macherey-Nagel™ NucleoSpin™ Tissue kit (Carlo Erba Reagents, Milan, Italy). The presence of clinically relevant carbapenemases, ESβLs, and AmpCs-encoding genes was detected by the Check-MDR CT103XL microarray kit (Check-Points Health B.V., Wageningen, Netherlands) followed by PCR of resistance genes including *bla*CTX-M, *bla*VEB, *bla*KPC, *bla*OXA-48-type, *bla*CMY, *bla*MOX, *bla*DHA, *aphA6, armA*, *aac*(6′*)-Ib-cr, qnrS*, and *qnrB*. The primer sequences, targeted genes, and amplicon sizes are listed in Supplementary Table 1. Bi-directional sanger sequencing was performed to characterize the resistance genes allelic variants. Amplicons were purified using a Wizard® SV Gel and PCR Clean-Up System (Promega, Madison, WI, USA), and the sequencing was performed by Microsynth Seqlab (Germany). The obtained sequences were analyzed with DNA Sequencher software (version 4.1.4) and the BLAST software (http://blast.ncbi.nlm.nih.gov/Blast.cgi).

### Molecular Typing

Pulsed-Field Gel Electrophoresis (PFGE) was carried out on 10 ESβL-producing *E. coli* isolates, chosen as representative, to investigate their genetic relatedness. The genomic DNA of each sample was digested with the *XbaI* restriction enzyme (45 U) and the genome fragments were separated on a CHEF mapper system (Bio-Rad Laboratories, Milan, Italy) at 14°C at 6 V/cm for 20 h with an initial pulse time of 0.5 s and a final pulse time of 30 s. Lambda 48.5 kb concatamers (New England BioLabs, Beverly, MA, USA) were used as a molecular size marker. Dendrogram of strains relatedness was obtained with Fingerprinting II version 3.0 software (Bio-Rad Laboratories, Milan, Italy) using UPGMA, according to the criteria described by Tenover et al. (1995). The dice correlation coefficient was used with a 1.0% of both position tolerance and optimization.

The phylogenetic groups (A, B1, B2, C, D, E, and F) were investigated for all *E. coli* isolates according to the Clermont scheme (Beghain et al., 2018).

The Multi-Locus Sequence Analysis was performed for *E. coli* and *K. pneumoniae* isolates according to the Achtman scheme (http://mlst.warwick.ac.uk/mlst/dbs/Ecoli) and the Pasteur Institute scheme (http://www.pasteur.fr/recherche/genopole/PF8/mlst/Kpneumoniae.html), respectively. PCR amplicons from housekeeping genes were purified using a Wizard® SV Gel and PCR Clean-Up System (Promega, Madison, WI, USA). Sequences were performed by the Microsynth Seqlab (Germany), and the analyses were performed with the DNA Sequencher software version 4.1.4.

Plasmid investigation (the determination of the Incompatibility groups) was accomplished by the PBRT KIT-PCR-based replicon typing (Diatheva, Fano, Italy), according to the manufacturer's instructions.

### Whole-Genome Sequencing (WGS)

The *E. coli* strain designated as C7 and C9-3 were subjected to DNA extraction using the Macherey-Nagel™ NucleoSpin™ Tissue kit (Carlo Erba Reagents, Milan, Italy). The extracted DNA was fragmented with Megaruptor 2 using the Hydropore-long (Diagenode, Belgium). Libraries' preparation of the fragmented DNA was accomplished according to the manufacturer's recommendation for microbial multiplexing with the Express kit 2.0. No size selection was performed during library preparation. Constructed libraries were sequenced using long-read sequencing technology on Sequel I (Pacific Biosciences, Menlo Park, California, USA).

### WGS Data Analyses

Genome assembly was performed with minimum seed coverage of 30×, using the “Microbial Assembly” pipeline offered by “SMRT Link v8.0.” Antibiotic resistance genes, plasmid replicons, virulence factors, and MLST were obtained through uploading assembled contigs to ResFinder (https://cge.cbs.dtu.dk/services/ResFinder/) (Bortolaia et al., 2020), PlasmidFinder (https://cge.cbs.dtu.dk/services/PlasmidFinder/) (Carattoli et al., 2014), Virulence factors database (http://www.mgc.ac.cn/VFs/) (Joensen et al., 2014), and MLST 2.0 (https://cge.cbs.dtu.dk/services/MLST/) (Larsen et al., 2012). The genome was annotated by the NCBI Prokaryotic Genome Annotation Pipeline (PGAP). Plasmids comparisons were assessed through the Blast Ring Image Generator (BRIG) application (http://brig.sourceforge.net). Genbank files were formatted and uploaded using the Sequin software (https://www.ncbi.nlm.nih.gov/Sequin/).

## Results

### Sampling Sites and Chemical Analyses Results

A total of 18 water samples were collected from distinct geographical locations, including canals (*n* = 7/18, 38.9%), streams and springs (*n* = 3/18, 16.6%), ponds (*n* = 2/18, 11.1%), small lake, sewer, and a WWTP (*n* = 1/18, 5.6%).

All the sampled waters showed a Ca-HCO_3_ facies with medium-low mineralization. The electrical conductivity resulted in the range between 170 and 650 μS/cm, while the pH values varied in a narrow range of values, between 7.4 and 7.8 (Table S2).

The waters showing less mineralization have a prevalent origin from irrigation water (samples 16, 17, and 18), while the waters with greater mineralization derive largely from the perched aquifer (samples 2, 4, 5, and 11).

The other waters are usually the result of a mixture of groundwater, surface water, and sewage wastewater.

The nitrate concentrations were generally on the order of a few mg/L for almost all waters sampled. Only three water samples showed higher nitrate concentrations (>5 mg/L) of anthropogenic origin (samples 4, 12, and 13).

COD values ranged from about 25 to almost 60 mg/L. Among these, the highest values, above 40 mg/L, were linked to water bodies fed by the suspended aquifer (samples 2, 4, 5, and 9) and were probably linked to the supply of run-off water from the vast agricultural expanses. The other COD values fell within the normally expected variability between waters of various origins.

### Bacterial Counts and Species Identification

**Figure 3:**
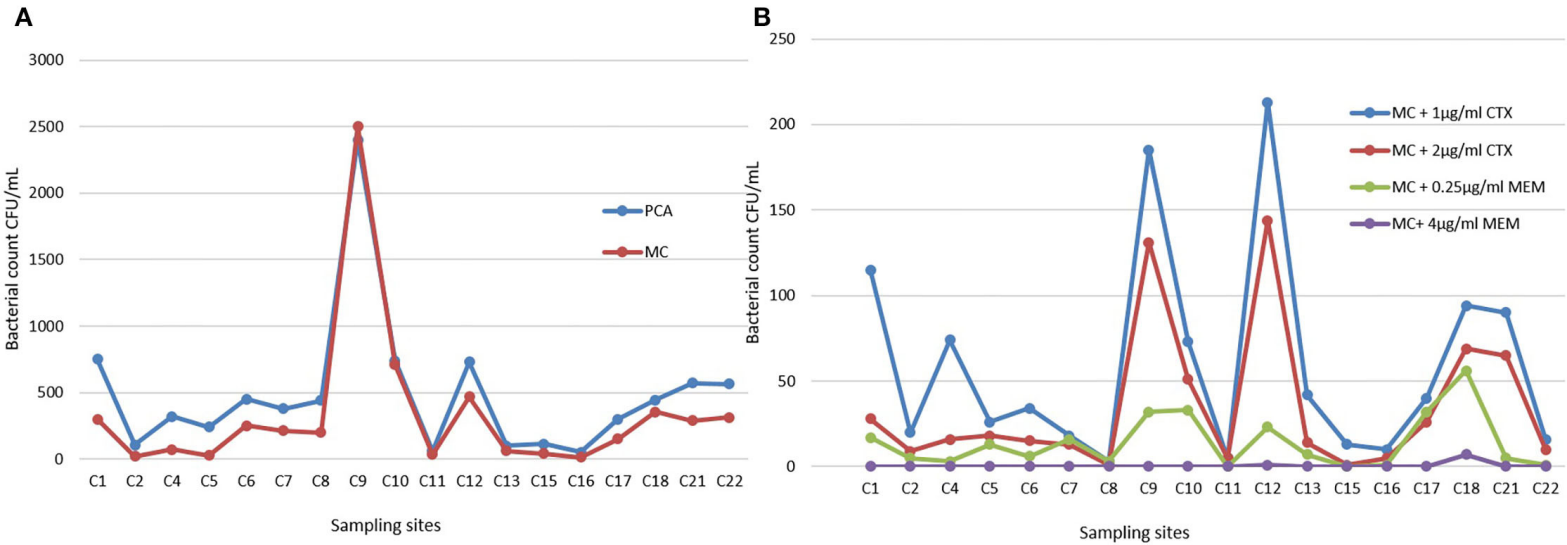
**(A)** Bacterial counts on Plate Count Agar (PCA) and MacConkey Agar (MC) plates in all sampling sites. **(B)** Selective MacConkey Agar plates supplemented with 1 μg/ml of Cefotaxime (CTX), 2 μg/ml of CTX, 0.25 μg/ml of Meropenem (MEM), or 4 μg/ml of MEM.

The range of bacteria CFU/ml identified after membrane filtration on PCA went from 51 to 2400 (Table S3). The highest bacterial load was detected in sampling site 9, a sewage wastewater that appeared to be a real outlier compared to other samples (Figure 3A). The same quantitative trend was obtained on MC plates, where only the Gram-negatives portion is considered (the calculation of the linear regression between PCA and MC shows a coefficient of determination *R*^2^ equal to about 0.95).

**Figure 4:**
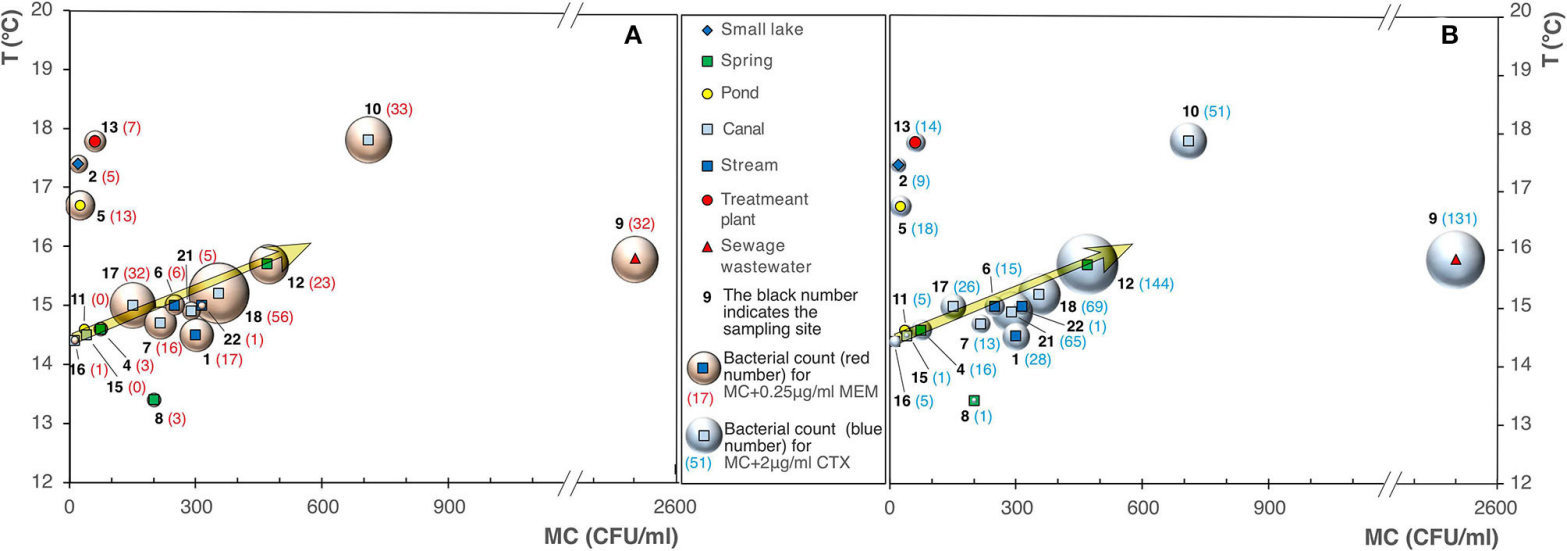
Bubble diagrams of bacterial counts on MacConkey (MC) vs. water temperature. **(A)** MC with addition of 0.25 μg/ml of meropenem (MEM); **(B)** with addition of 2 μg/ml of cefotaxime (CTX).

The analysis of the data highlighted an increase in the bacterial count on MacConkey agar (MC), and MC added with 0.25 μg/ml of MEM (Figure 4A), and 2 μg/ml of CTX (Figure 4B), as the waters temperature increases.

The sites 9, 12, 1, 18, 21, 22, and 4 already emerged as highly productive on PCA/MC, and appeared characterized by noteworthy bacterial counts also on MC added of β-lactams, as shown in Figure 3B.

**Figure 5:**
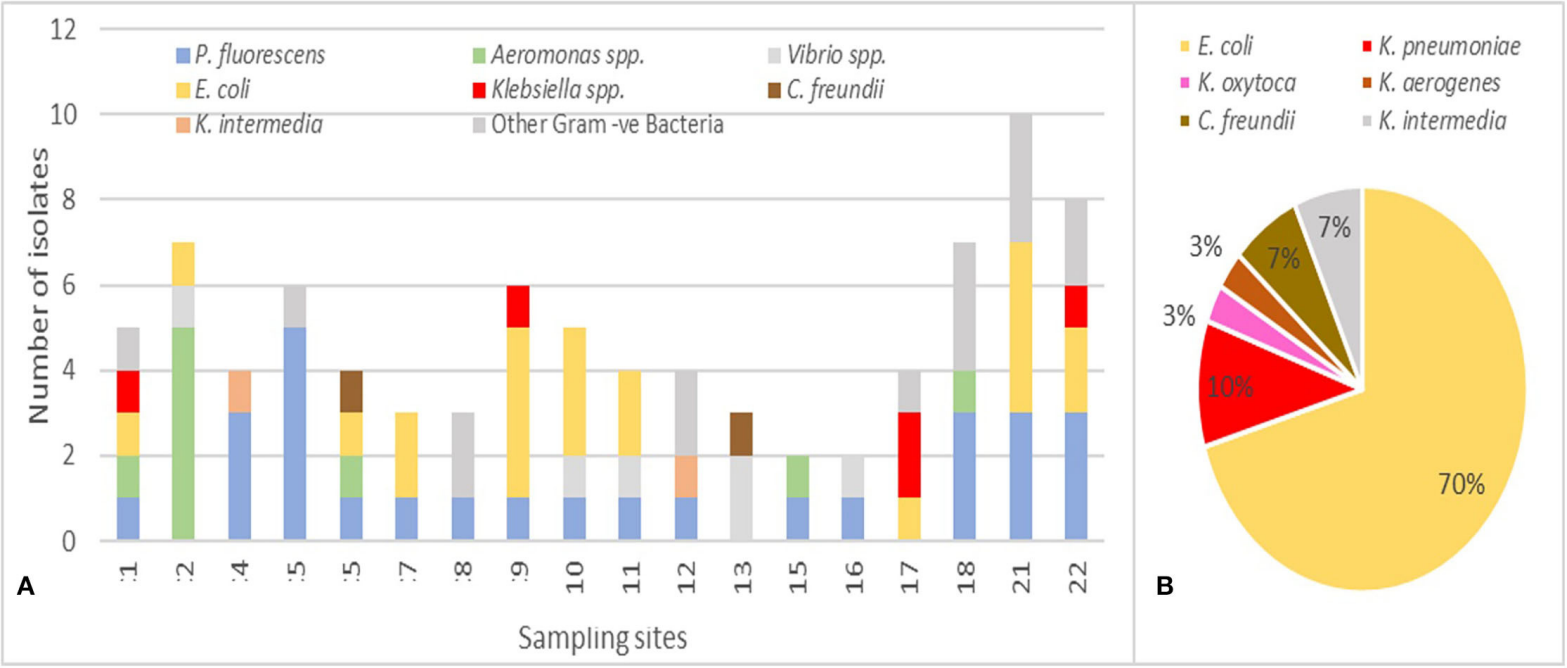
**(A)** Distribution of the 87 bacterial species identified in the 18 sampling sites. **(B)** Distribution of *Enterobacterales* spp. (values are in %, *n* = 30) with *E. coli* representing 70% of the species.

Based on the differences in the morphological aspect, 87 Gram-negative isolates, obtained on MC + CTX and/or MC + MEM, were collected and subjected to bacterial identification and antibiotic susceptibility determination by semi-automated MicroScan autoSCAN-4 system (Figure 5A). The 34% (*n* = 30/87) of the isolates were *Enterobacterales*, 70% (*n* = 21/30) were *E. coli*, 16.6% (*n* = 5/30) *Klebsiella* spp. (3 *K. pneumoniae*, 1 *K. oxytoca*, and 1 *K. aerogenes*), 6.7% (*n* = 2/30) *Citrobacter freundii*, and 6.7% (*n* = 2/30) *Kluyvera intermedia* (Figure 5B). The remaining isolates included *Pseudomonas fluorescens* (31%; *n* = 27/87), *Aeromonas* spp. (11.5%; *n* = 10/87), *Vibrio* spp. (5.7%; *n* = 5/87), and other Gram-negative bacteria (16.1%; *n* = 14/87). The most represented species *E. coli* was identified in 10/18 sites, being absent in 4, 5, 8, 12, 13, 15, 16, and 18. *Klebsiella* spp., the second species for prevalence detected on MC + CTX and/or MC + MEM, was identified in 4/18 (indicated as 1, 9, 17, and 22) sampling sites.

### Antibiotics Resistance Profiles and Phenotypic Tests

**Figure 6:**
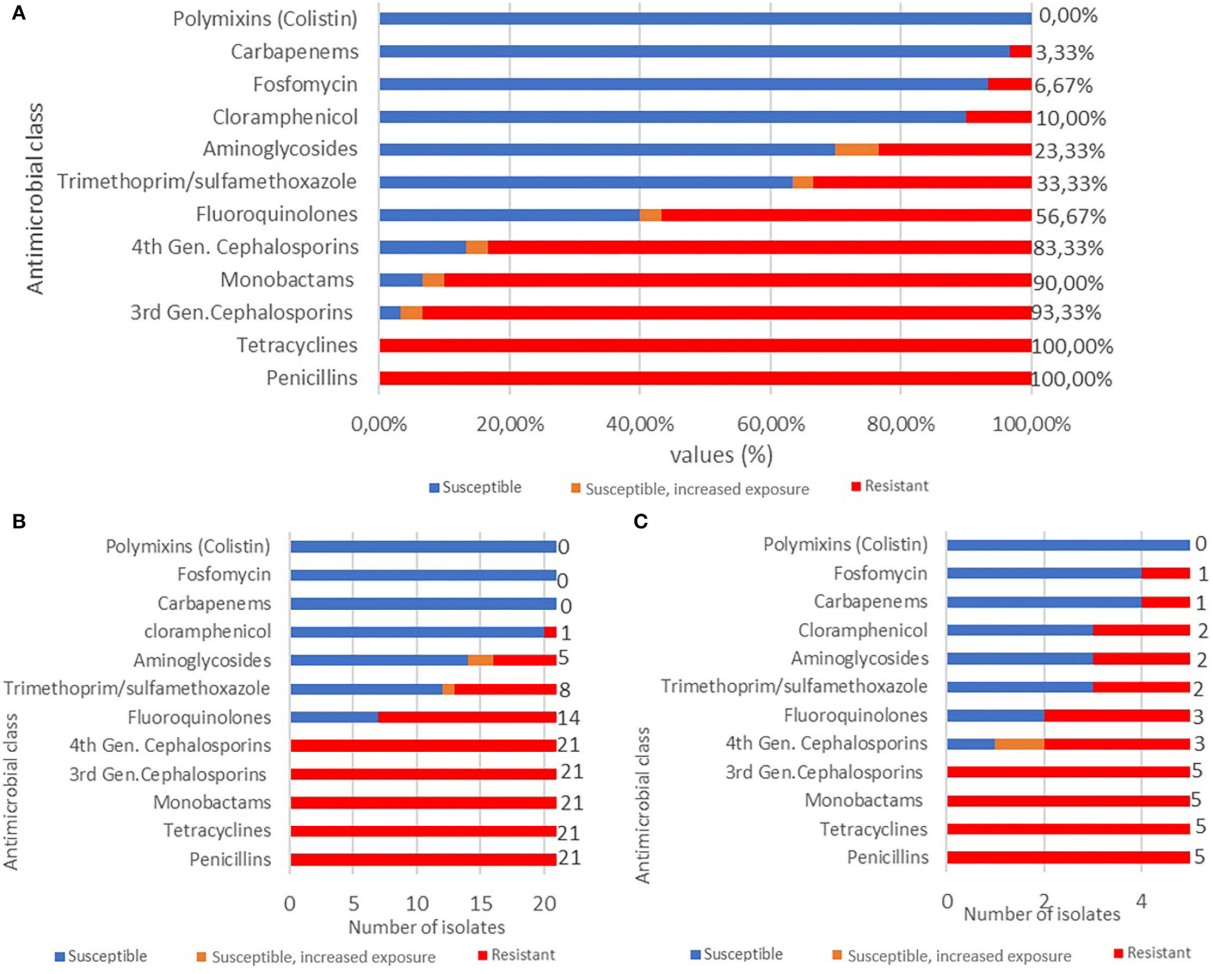
**(A)** Antimicrobial resistance profile of *Enterobacterales* (*n* = 30) isolates to 10 different antimicrobial classes. **(B)** Antimicrobial resistance profile of MDR *E. coli* (*n* = 21) isolates to 10 antimicrobial classes. **(C)** Antimicrobial resistance profile of *Klebsiella* spp. (*n* = 5) isolates 10 antimicrobial classes.

Antimicrobial susceptibility profiles of the 30 *Enterobacterales* isolates showed resistance to penicillins (100%), tetracyclines (100%), 3GCs (93.3%), monobactams (90%), fourth generation cephalosporins 4GCs (83.33%), fluoroquinolones (56.7%), trimethoprim/sulfamethoxazole (33.3%), aminoglycosides (23.3%), chloramphenicol (10%), fosfomycin (6.7%), and carbapenems (3.3%) (Figure 6A). Remarkably, 61.9% of *E. coli* isolates (*n* = 13) exhibited a MDR phenotype showing complete resistance to penicillins, 3GCs, 4GCs, tetracyclines, fluoroquinolones (*n* = 14, 66.7%), trimethoprim/sulfamethoxazole (*n* = 8, 38%), aminoglycosides (*n* = 5, 23.8%), and chloramphenicol (*n* = 1, 4.76%) (Figure 6B).

*Klebsiella* spp. strains were 100% (*n* = 5) resistant to 3GCs, tetracyclines, and monobactams, 60% (*n* = 3) to 4GCs and fluoroquinolones, 40% (*n* = 2) to trimethoprim/sulfamethoxazole, aminoglycosides, and chloramphenicol, and 20% (*n* = 1) to fosfomycin and carbapenems (Figure 6C).

A high proportion of the enterobacteria (80%; *n* = 24/30) were confirmed as ESβL-producers, showing a synergic effect between clavulanic acid and 3GCs/4GC. All *E. coli* (*n* = 21) and *K. pneumoniae* (*n* = 3) isolates exhibited ESβL-producing phenotypes and were further investigated. In addition, one *K. pneumoniae* isolate showed a carbapenemase phenotype.

### Molecular Characterization of ESβLs and Carbapenemases Genes

**Figure 7:**
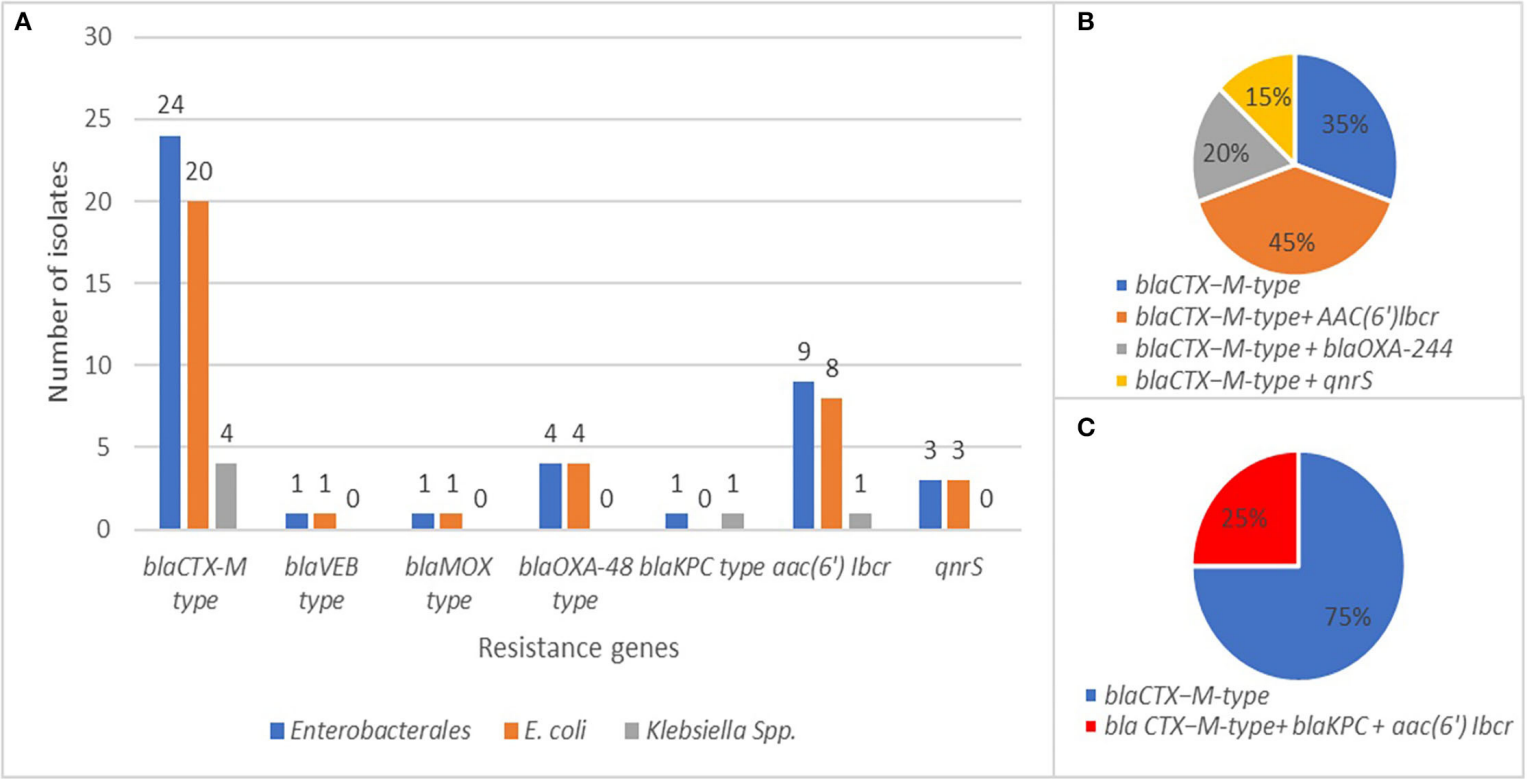
**(A)** Frequency and distribution of resistance genes in *E. coli* and *K. pneumoniae* isolates. **(B)** Co-occurrence of *bla*CTX-M with other resistance genes in *E. coli* isolates. **(C)** Co-occurrence of *bla*CTX-M with other resistance genes in *K. pneumoniae* isolates.

Molecular analysis (PCR and sequencing) revealed the presence of at least one β-lactamase gene in each ESβL-producing isolate phenotypically detected. The *bla*CTX-M-type, *bla*OXA-244, *bla*KPC-2, *bla*MOX-2, and *bla*VEB-1 genes were detected in 80% (*n* = 20 *E. coli*; *n* = 4 *Klebsiella* spp.), 13.3% (*n* = 4 *E. coli*), 3.3% (*n* = 1 *K. pneumoniae*), 3.3% (*n* = 1 *E. coli*), and 3.3% (*n* = 1 *E. coli*) of *Enterobacterales* isolates, respectively (Figure 7A). The *bla*CTX-M-type were the most common ARGs identified, especially *bla*CTX-M-group-1.

Fluoroquinolones-resistant isolates harbored mainly *aac(6*′*)-Ib-cr* and/or *qnrS*. Nevertheless, isolates encoding *qnrS* were also identified among ciprofloxacin and levofloxacin-susceptible strains.

However, some isolates harboring *aac(6*′*)-Ib-cr* showed reduced susceptibility to amikacin. In five cases (*n* = 4 *E. coli*; *n* = 1 *K. pneumoniae*), the *bla*CTX-M-type gene coexisted with other two β-lactamase determinants, *bla*OXA-244 (*n* = 4 *E. coli*) (Figure 7B) and *bla*KPC-2 (*n* = 1 *K. pneumoniae*) (Figure 7C). The co-occurrence of *bla*CTX-M with *aac(6*′*)-Ib-cr* or *qnrS* genes was found in 45 and 15% of *Enterobacterales* isolates, respectively (Figure 7, Table S4 and Table 1). One *E. coli* isolate carried both *bla*VEB-1 and *bla*MOX-2 genes, as shown in Table S4 and Table 1.

**Table 1:** Phenotypic/molecular characteristics of ESβL/carbapenemases-producing *E. coli* and *Klebsiella* spp. isolates recovered from the 18 sampling sites.

**Sample name** ^a^	**Feature** ^b^	**Phylogroup**	**MLST** ^e^	**Antimicrobial Resistance** ^c^	**Resistance genes for** ^d^		
					**β-lactams**	**Quinolones**	**Aminoglycosides**
C1 *E. coli*	ESβL +	B2	NT	AMP, AMC, PIP, ATM, FEP, CTX, CAZ, CXM, CIP, LEV, GEN, TOB, TET, TMS	*bla*CTX-M-type	*aac(6*′*)-Ib-cr*	*aac(6*′*)-Ib-cr*
C1 *K. pneumoniae*	ESβL + Carba R+	/	ST258	AMP, AMC, PIP, ATM, CLO, FEP, CTX, CAZ, CXM, CIP, LEV, AK, GEN, TOB, ERT, IMI, MER, TZP, TET	*bla*KPC-2 *bla*CTX-M-type	*aac(6*′*)-Ib-cr*	*aac(6*′*)-Ib-cr*
C2 *E. coli*	ESβL +	B2	NT	AMP, AMC, PIP, ATM, FEP, CTX, CAZ, CXM, CIP, LEV, AK(I) TET	*bla*CTX-M-27	*aac(6*′*-)Ib-cr*	*aac(6*′*)-Ib-cr*
C6 *E. coli*	ESβL +	B2	NT	AMP, PIP, ATM, FEP, CTX, CAZ, CXM, CIP, LEV, TET, TMS	*bla*CTX-M-type	*aac(6*′*)-Ib-cr*	–
C7 *E. coli* (WGS)	ESβL +	B2	ST131	AMP, AMC, PIP, ATM, FEP, CTX, CAZ, CXM, CIP, LEV, TZP	*bla*OXA-244 *bla*CTX-M-15	Chromosomal Mutations	*aadA5*
C7-2 *E. coli*	ESβL +	Unknown	NT	AMP, PIP, ATM, FEP, CTX, CAZ, CXM, TET	*bla*CTX-M-type	–	–
C9 *K. oxytoca*	ESβL –	/	NT	AMP, AMC, PIP, ATM, CTX, CAZ, CXM, TZP, TOB, TET, TMS	not detected	–	–
C9-1 *E. coli*	ESβL +	D	NT	AMP, PIP, ATM, FEP, CTX, CAZ, TET, AK(I), GEN(I)	*bla*CTX-M-type	–	–
C9-2 *E. coli*	ESβL +	B2	ST131	AMP, PIP, ATM, FEP, CTX, CAZ, TET	*bla*OXA-244 *bla*CTX-M-type	–	–
C9-3 *E. coli* (WGS)	ESβL +	B2	ST131	AMP, AMC, PIP, ATM, FEP, CTX, CAZ, CLO, CIP, LEV, TZP, TET, TMS	*bla*OXA-244 *bla*CTX-M-15	Chromosomal Mutations	*aadA5*
C9-4 *E. coli*	ESβL +	B2	ST131	AMP, AMC, PIP, ATM, FEP, CTX, CAZ, CIP, LEV, TZP, TET, TMS	*bla*OXA-244 *bla*CTX-M-type	–	–
C10-1 *E. coli*	ESβL +	A	NT	AMP, AMC, PIP, ATM, FEP, CTX, CAZ, CXM, CIP, LEV, AK(I), TZP(I), GEN, TOB, TET, TMS	*bla*CTX-M-type	*aac(6*′*)-Ib-cr*	*aac(6*′*)-Ib-cr*
C10-2 *E. coli*	ESβL +	D	ST648	AMP, AMC, PIP, ATM, FEP, CTX, CAZ, CXM, COL, CIP, LEV, AK(I), GEN, TOB, TET	*bla*CTX-M-type	*aac(6*′*)-Ib-cr*	*aac(6*′*)-Ib-cr*
C10-3 *E. coli*	ESβL +	D	ST648	AMP, AMC, PIP, ATM, FEP, CTX, CAZ, CXM, COL, CIP, LEV, AK(I), GEN, TOB, TET	*bla*CTX-M-type	*aac(6*′*)-Ib-cr*	*aac(6*′*)-Ib-cr*
C11-1 *E. coli*	ESβL +	A	ST23	AMP, PIP, ATM, FEP, CTX, CAZ(I), COL, TET	*bla*CTX-M-1	–	–
C11-2 *E. coli*	ESβL +	A	ST23	AMP, PIP, ATM, FEP, CTX, CXM, CAZ(I), TET	*bla*CTX-M-1	–	–
C17 *K. pneumoniae*	ESβL +	/	NT	AMP, PIP, ATM, FEP, CTX, CAZ(I), CXM, CIP, TET	*bla*CTX-M-type	*qnrS*	–
C17 *E. coli*	ESβL +	A	NT	AMP, PIP, ATM, FEP, CTX, CXM, CIP, LEV, TET, TMS	*bla*CTX-M-type	*qnrS*	–
C17-2 *K. pneumoniae*	ESβL +	/	NT	AMP, PIP, ATM, FEP, CTX, CAZ, CXM, CIP, TET, TMS	*bla*CTX-M-type	*qnrS*	–
C21 *E. coli*	ESβL +	B2	ST131	AMP, PIP, ATM, FEP, CTX, CAZ, CXM, CIP, LEV, TET	*bla*CTX-M-14	–	–
C21-2 *E. coli*	ESβL +	B1	NT	AMP, PIP, ATM, FEP, CTX, CAZ, CXM, TET, AK(I), TOB, TMS	*bla*CTX-M-type	*aac(6*′*)-Ib-cr*	*aac(6*′*)-Ib-cr*
C21-3 *E. coli*	ESβL +	B2	ST131	AMP, PIP, ATM, FEP, CTX, CAZ, CIP, LEV, TET, TMS(I)	*bla*CTX-M-type	–	–
C21-4 *E. coli*	ESβL +	D	NT	AMP, AMC, PIP, ATM, FEP, CTX, CAZ(I), CXM, CIP, LEV, TET	*bla*CTX-M-gr9	–	–
C22 *K. aerogenes*	ESβL –	/	NT	AMP, PIP, ATM, FEP, CTX, CAZ, CLO, TZP, TET	*bla*CTX-M neg	–	–
C22 *E. coli*	ESβL +	Negative for all	NT	AMP, PIP, ATM, FEP, CTX, CAZ, CXM, TET	*bla*CTX-M-type	–	–
C22-2 *E. coli*	ESβL +	D	NT	AMP, PIP, ATM, FEP, CTX, CAZ, CXM, CIP, LEV, TET	*bla*VEB-1 *bla*MOX-2	–	–

a *Sample was subjected to whole-genome sequencing*.

b *ESβL, Extended spectrum β-lactamase; CARBA R, carbapenem-resistant; LAC-, lactose non-fermenter*.

c *AMC, amoxicillin/clavulanic acid; AMP, ampicillin; PIP, piperacillin; ATM, aztreonam; FEP, cefepime; CTX, cefotaxime; CAZ, ceftazidime; CXM, cefuroxime; CIP, ciprofloxacin; LEV, levofloxacin; TZP, piperacillin-tazobactam, AK, amikacin; GM, gentamicin; TOB, tobramycin; ERT, ertapenem; IMI, imipenem; MEM, meropenem; TET, tetracycline; TMS, trimethoprim/sulfamethoxazole*.

d *-, isolate was not checked for the presence of resistance genes due to phenotypic susceptibility*.

e *NT, not tested*.

### Molecular Typing

**Figure 8:**
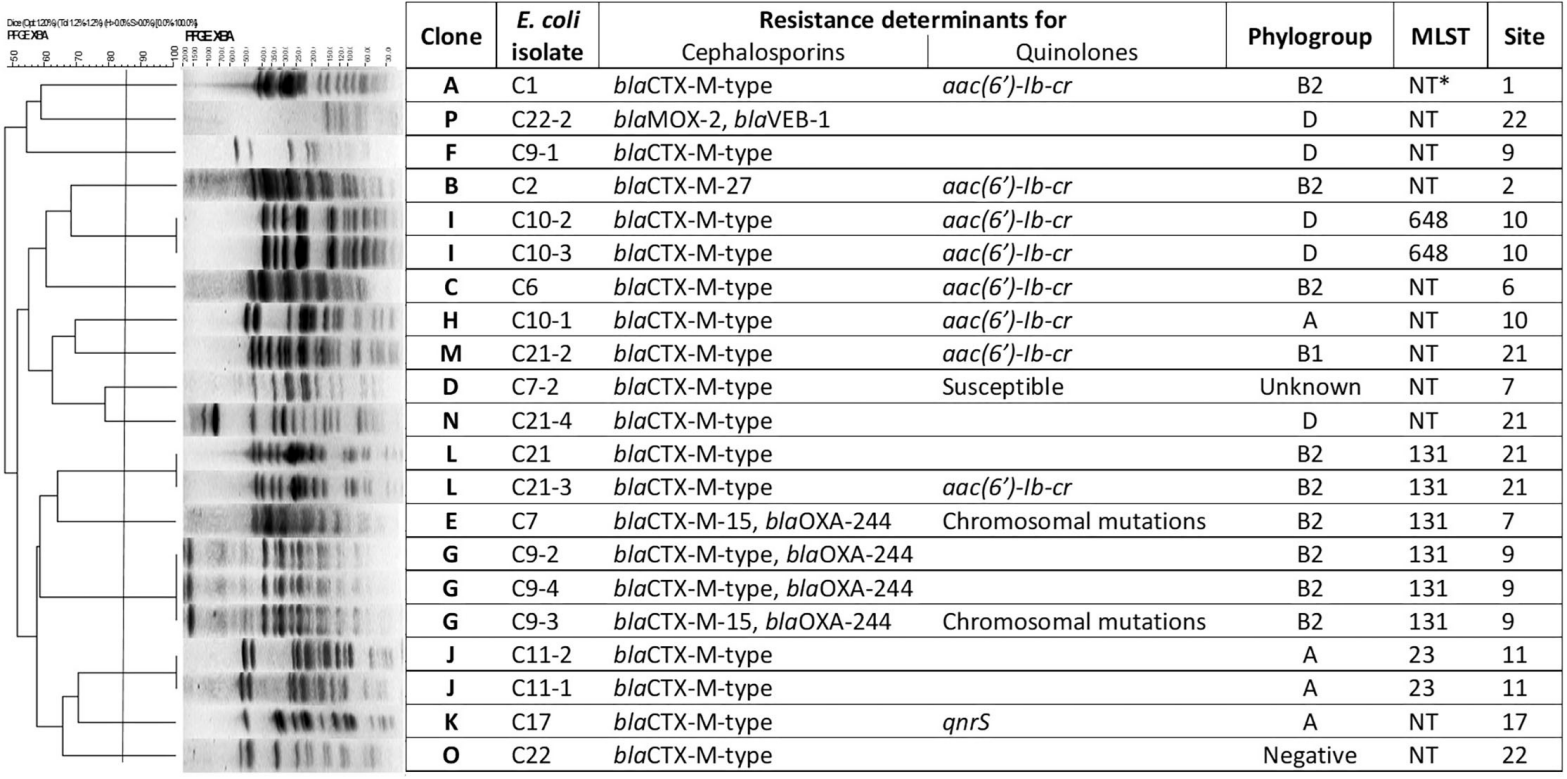
Cluster analysis of the 21 ESβL-producing *E. coli* isolates. *NT, Not Tested.

PFGE analysis of all the 21 ESβLs-producing *E. coli* isolates showed clonal heterogeneity, showing 16 different pulsotypes (Figure 8). Among the 21 ESβLs-producing *E. coli* strains, the predominant phylogenetic group identified was B2 (*n* = 9/21; 42.9%), followed by D (*n* = 6/21; 28.6%), A (*n* = 4/21; 19%), and B1 (*n* = 1/21; 4.8%). One isolate (4.8%) resulted negative for all the detectable phylogroups. The two OXA-producing *E. coli* isolates, C7 and C9-3, collected in different sampling sites and not clonally related by PFGE, resulted the CTX-M-15 and OXA-244 enzyme co-producers. More interestingly, the above strains belonged to the pandemic high-risk clone ST131.

The C1 *K. pneumoniae* strain harboring the *bla*KPC-2, *bla*CTX-M-type, and *aac(6*′*)-Ib-cr* resistance genes belonged to the hyper-epidemic clone ST258.

### Whole-Genome Sequencing of *E. coli* C7 and C9-3 Isolates

To better characterize the *bla*OXA-244-producing strains, Whole-Genome Sequencing (WGS) was performed. The C7 *E. coli* genome (GenBank accession num.: CP059279:CP059280) includes a chromosome of 4,998,812 bp and a plasmid of 141,169 bp. The *E. coli* C9-3 genome (GenBank Accession num.: CP059281:CP059282) presents a chromosome of 4,999,584 bp and a plasmid of 140,350 bp. Both strains belonged to the ST131 and phylogroup B2, and showed the O16:H5 serotype and the fimbrial variant fimH41.

**Figure 9:**
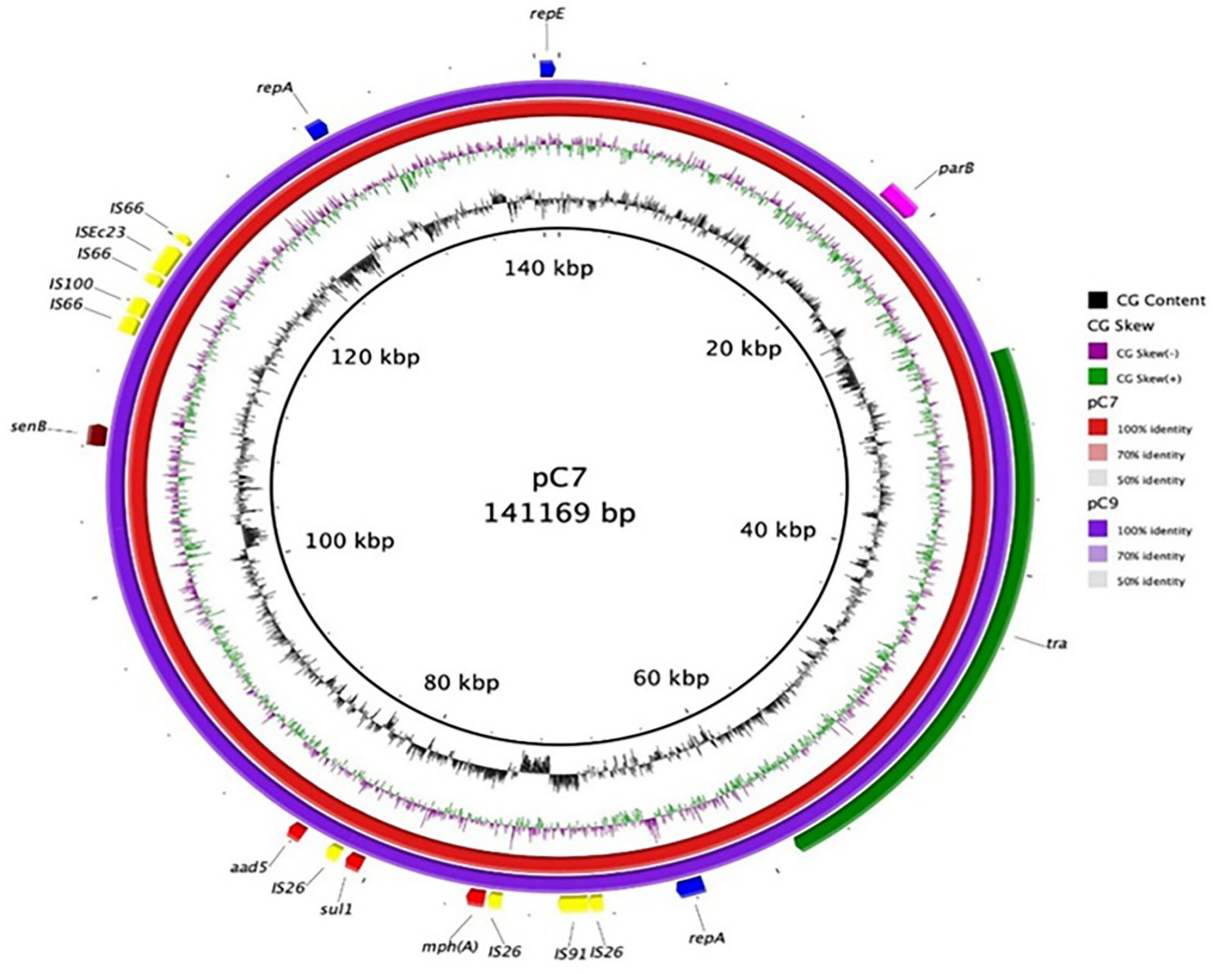
Plasmid map of C7 and C9-3 *E. coli*. Each ring represents the nucleotide sequence of the plasmids of the two *E. coli* isolates as indicated by the color code. Gene annotation of the reference plasmid pC7 is shown in the outer ring. Colored arrows indicate the genes, *mph(A)* (macrolide 2′-phosphotransferase), *sul1* (dihydropteroate synthase), *senB* (enterotoxin), *repA* (regulatory protein), *repE* (replication initiation protein), *parB* (centromer binding protein), and *aad5* (aminoglycoside adenyl transferase).

The resistance determinants and virulence factors were the same for both strains (Table S5). Interestingly, both isolates harbored the β-lactamase genes *bla*CTX-M-15 and *bla*OXA-244 on the chromosome, in addition to *mdf(A)* gene, usually found in MDR isolates and encoding a multidrug resistance efflux pump which confers resistance to many molecules such as erythromycin, chloramphenicol, fluoroquinolones, and to a much lesser extent to neomycin and kanamycin (Table S5) (Edgar and Bibi, 1997; Ong et al., 2020). In these two isolates, many resistance genes were harbored on a IncFIA plasmid, including resistance genes for macrolides (*erm(B)*), aminoglycosides (*aadA5*), sulphonamides (*sul1*), and trimethoprim (*dfrA17*) (Table S5 and Figure 9).

**Figure 10:**
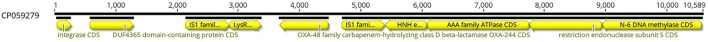
Schematic representation of the 10,578 bp genetic segment representing the genetic environment of chromosomally encoded *bla*OXA-244 harbored in the IS1-made composite transposons.

WGS analysis showed that, for both C7 and C9-3 *E. coli* strains, the *bla*OXA-244 gene was part of a composite transposon chromosomally located and bracketed by one copy of the insertion sequence IS1 showing the same direction (Figure 10). This composite transposon included two open reading frames encoding the *bla*OXA-244 and *lysR* genes, a transcriptional regulator with 98% identity to the acetyl-coenzyme A carboxylase multifunctional enzyme, AccADC39. Moreover, *bla*CTX-M-15 was also located on the chromosome with the IS1380-like element ISEc9 family transposase.

Both C7 and C9-3 *E. coli* strains harbored on their chromosome multiple virulence factors; the majority of which are typically exhibited by ST131 isolates, including adhesins-siderophore receptor (*iha*), toxins (*sat, usp, hlyE/clyA*), siderophores (*iucD*), and autotransporter systems (*tsh*), group 2 capsule (kpsMII-k2). Both strains showed the virotype C2, according to the scheme proposed by Dahbi (Dahbi et al., 2014). The C7 and C9-3 strains lack two virulence classes involved in adhesion: a fimbrial adhesin AFA-I and Curli fibers found in other ST131 *E. coli* O25b:H4 sub-clones. Only the *senB* gene, coding for the enterotoxin SenB/TieB, was located on the IncFIA plasmid (Figure 9).

### Comparison of the *bla*OXA-244 Genetic Environment

The genetic environment of *bla*OXA-244 was investigated; a 10,578 bp genetic segment was *in silico* extracted (Figure 10). Upstream of the transposon harboring *bla*OXA-244, genes coding for integrase, and DUF4365 domain were found. Furthermore, downstream of the transposon genes coding for HNH endonuclease, AAA family ATPase, restriction endonuclease subunit S, and N-6 DNA methylase were found. This segment was blasted against the NCBI database; C9-3 and C7 had 100% sequence identity and query with 10 submissions from the same study conducted in the Netherlands in 2021 (CP068813, CP086616, CP086618, CP086620, CP086622-CP086626, and CP087377). All the genomic hits belonged to *E. coli* strains isolated from clinical samples. Another two genomes had similar high scores (sequenced identity 99.99 and 100% sequence query) and they belonged to one *E. coli* strain (52148CZ) isolated in the Czech Republic in 2021 from a clinical sample (Chudejova et al., 2021) and to one *E. coli* strain (28Eco12) isolated in Colombia in 2019 from a clinical sample. The high number of blast hits of the same 10 Kbp segments suggest that the transposon harboring *bla*OXA-244 is inserted in the same position in *E. coli* isolates belonging to different STs. This suggests that the transposon has a hotspot for insertion in this position, more specifically within the gene coding for HNH endonuclease. The gene seems to be truncated whenever the transposon is inserted in this region while having a complete gene in other positions when compared with other strains.

## Discussion

This study describes the occurrence of CTX-M-type ESβLs and KPC- and OXA-48-type carbapenemases among *Enterobacterales* collected from the surface water in Pavia. This study is the first to report (i) the presence of an ST131 *E. coli* strain co-harboring *bla*CTX-M-15 and *bla*OXA-244 genes and (ii) the presence of a KPC-2-producing ST258 *K. pneumoniae* from surface and groundwaters of Pavia, Northern Italy.

Along the Vernavolino and Navigliaccio canals watercourse, from upstream to downstream of the Pavia urban area, an increase in the Gram-negatives bacterial load was evident on MacConkey agar plates. The same trend was also observed in the case of MacConkey agar added with the two antibiotics CTX and MER. In the Roggia Vernavola watercourse, the Gram-negatives bacterial load remained constant in all the three sampling sites (1, 6, and 22). Interestingly, in this case, the non-wild-type Gram-negative bacterial load was higher in the upstream sites, and this is probably because the Roggia Vernavola watercourse originates in another urban area (San Genesio ed Uniti).

The high density of Gram-negative bacteria contamination and high rates of non-susceptibility to CTX and MER were correlated and observed in the most urbanized areas; MDR phenotypes were observed particularly near sewage discharges. Moreover, both C7 and C9-3 *bla*OXA-244-*E. coli* strains were found inside the urban area (sites 7 and 9). All of this shows how local anthropogenic activities can directly affect water quality.

**Figure 11:**
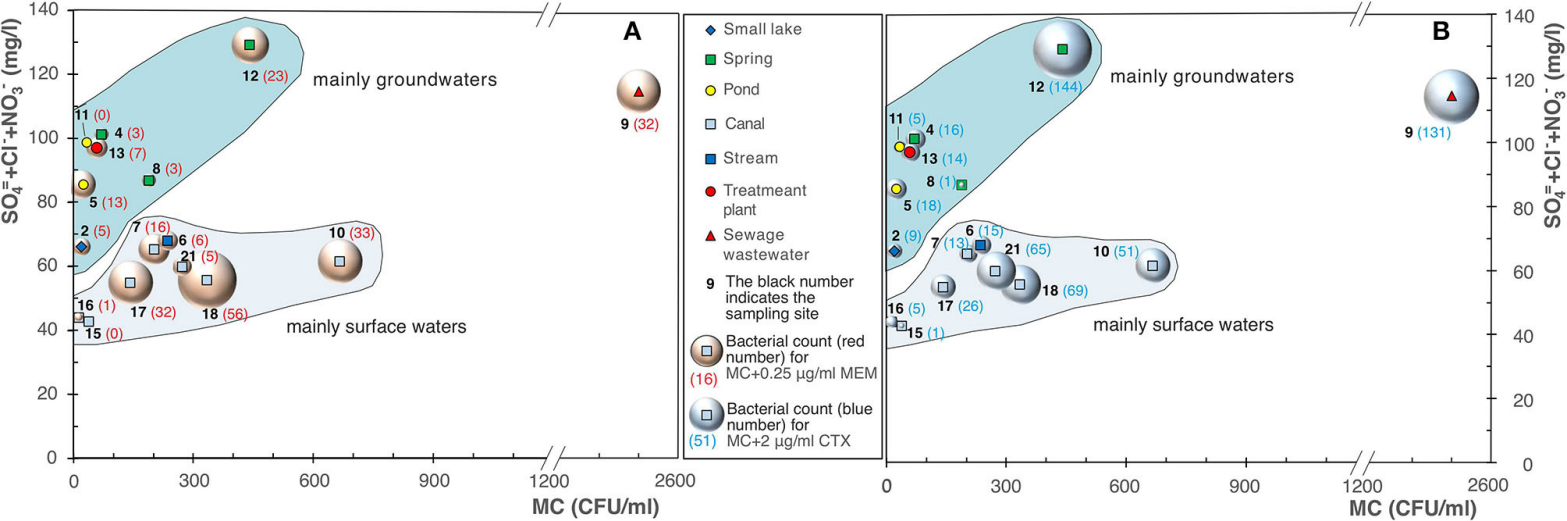
Bubble diagrams of bacterial counts on MacConkey agar (MC) vs. ${\text{SO}}_{4}^{\text{=}}+{\text{Cl}}^{-}+{\text{NO}}_{3}^{-}$. **(A)** MC with addition of 0.25 μg/ml of meropenem (MEM); **(B)** MC added with 2 μg/ml of cefotaxime (CTX).

Our results showed a substantial difference, with the only exception of site 9 (sewage wastewater), among surface waters (canals and streams) and groundwaters (springs, pond, and small lake, considering the latter receives a substantial water contribution from the groundwaters). In the groundwaters, although they showed evident signs of an anthropic impact due to the presence of substances such as sulfates, nitrates, and chlorides of superficial origin (Pilla et al., 2006), we detected a modest increase in the bacterial load on MacConkey agar and on MacConkey agar added with 0.25 μg/ml of MEM (Figure 11A) and 2 μg/ml of CTX (Figure 11B). Site 12 (a spring) showed a very high bacterial count and this can be due to the fact it receives water from sewers. Overall, the results highlighted that the groundwaters showed a lower bacterial load on MacConkey agar compared to the surface waters (Navigliaccio, Vernavola, and Vernavolino).

An unexpected and positive datum was the water microbiological quality, in terms of low bacterial load, found at the site of the Pavia treatment plant, an index of the aforementioned system's good functioning.

The high occurrence of CTX-M-type ESβL-producing bacteria in the surface and groundwaters of Pavia is worrisome, reaching a critical level in the surface waters. It is of particular concern that all ESβLs-producing isolates detected in this study were resistant to at least two classes of antibiotics including fluoroquinolones, aminoglycosides, and sulfonamides. Generally, cross-resistance can be explained by the fact of having *bla* genes located on conjugative plasmids which also harbor genes conferring resistance to other antibiotic classes such as fluoroquinolones and aminoglycosides (Gniadkowski, 2001). Of greater concern is the occurrence of *bla*OXA-244 in ESβL-producing *E. coli*, which are not susceptible to carbapenems, the last resort to treat infections caused by ESβL-producing strains (Lutgring and Limbago, 2016), in surface and groundwaters. A recent ECDC risk assessment indicated a pan-European problem, with a high risk of spread of OXA-244-producing *E. coli*, given the rapid and simultaneous increase in multiple countries between 2013 and 2020 (European Centre for Disease Prevention and Control (ECDC), 2020). The ECDC highlighted how the transmission of OXA-244-producing *E. coli* strains in the community could represent a real risk for public health contributing to the loss of carbapenems as options for treatment of *E. coli* infections. These two *bla*OXA-244 harboring *E. coli* isolates, showing carbapenems MICs values within the clinical susceptibility (although higher than ECOFF value), were found in site 7, inside the urban area between houses; and site 9, a sewer drain. These sites are reachable by wild animals, companion animals, and humans indicating the potential of *bla*OXA-244 silent dissemination, taking also into account that its real spread may be underestimated due to its difficult detection.

Moreover, *bla*OXA-244-producing *E. coli* and many MDR *E. coli* isolates detected belonged to the worldwide pandemic multi-resistant *E. coli* strain B2:ST131 that is strongly associated with severe infections in humans and animals (Rogers et al., 2011).

The *bla*OXA-244-harboring *E. coli* isolates found in this study belonged to the ST131-O16:H5 fimH41 clonal subgroup, whose occurrence is still limited in comparison to the highly virulent ST131 O25b:H4 *E. coli* (Dahbi et al., 2014). However, a murine infection model showed that ST131 O16:H5 *E. coli* belonging to the fimH41/virotype C, as the strains here studied, showed a virulence and lethality equivalent to that of ST131 O25b:H4 *E. coli* underlying its relevance (Dahbi et al., 2014; Mora et al., 2014). Moreover, an association between ST131-O16:H5 strains and pyelonephritis in men and reproductive-age women has been reported (Mora et al., 2014). Remarkably, CTX-M-14-producing O16:H5 isolates had been described, but there are no reports, to date, about the co-production of the *bla*CTX-M-15 and the *bla*OXA-244 genes in such clone, to the best of our knowledge. The silent dissemination of such isolates harboring *bla*OXA-244 and *bla*CTX-M-15 among other resistance determinants to other classes of antibiotics in the community may contribute to the loss of carbapenems as options for treatment of serious *E. coli* infections.

Moreover, the co-occurrence of *bla*KPC-2 and *bla*CTX-M genes in ST258 *K. pneumoniae* strains in the surface water is particularly worrisome, since this lineage is a frequent cause of hospital-associated outbreaks and a major contributor to the global spread of carbapenemases (Pitout et al., 2015). The *bla*KPC-2-harboring *K. pneumoniae* isolates have already been reported in the surface water in the Pavia region but are linked to ST307 (Caltagirone et al., 2017). The occurrence of such carbapenem-resistant strain from surface water highlights the need for further studies to understand the origin of this contamination and to plan containment actions.

## Conclusions

In conclusion, the detection of *bla*OXA-244-harboring ST131 *E. coli*, in addition to the presence of KPC-2 ST258 *K. pneumoniae* and ESβL-producing *E. coli* in surface and groundwaters, sites reachable by wild animals, companion animals, and humans, represent a significant threat for the spread of MDR bacteria in the community and should not be underestimated.

## Data Availability Statement

The datasets presented in this study can be found in online repositories. The names of the repository/repositories and accession number(s) can be found in the article/Supplementary Material.

## Author Contributions

Conceptualization: RM, GP, and RS. Formal analysis and interpretation of the data: AA, AP, AM, FM, VMM, IB, MS, GP, JH, and RS. Writing—original draft preparation: AA and AP. Writing—review and editing: AP, GP, RS, and RM. Supervision: GP and RM. All authors have read and agreed to the published version of the manuscript.

## Funding

This study was supported by the Charles University Research Fund PROGRES (project number Q39) and by project CZ.02.1.01/0.0/0.0/16_019/0000787 Fighting Infectious Diseases, provided by the Ministry of Education Youth and Sports of the Czech Republic.

## Conflict of Interest

The authors declare that the research was conducted in the absence of any commercial or financial relationships that could be construed as a potential conflict of interest.

## Publisher's Note

All claims expressed in this article are solely those of the authors and do not necessarily represent those of their affiliated organizations, or those of the publisher, the editors and the reviewers. Any product that may be evaluated in this article, or claim that may be made by its manufacturer, is not guaranteed or endorsed by the publisher.
