# Supplementary material for: OXA-244-Producing ST131 Escherichia coli From Surface and Groundwaters of Pavia Urban Area (Po Plain, Northern Italy)
Source: Front Microbiol. 2022 Jun 9;13:920319. doi: 10.3389/fmicb.2022.920319 (PMC9225575; doi:10.3389/fmicb.2022.920319)
Supplement: Supplementary file 1 [file Data_Sheet_1.docx]

# Supplementary material

**Supplementary Table 1.** Primers sequences, annealing temperatures, and PCR product size

| **Resistance determinant** | **Primer Sequence** | **Annealing temperature (^o^C)** | **Amplicon size (bp)** | **Reference** |
| --- | --- | --- | --- | --- |
| *bla*CTX-M-type | FW: 5′-ATGTGCAGYACCAGTAARGT-3′ | 50 | 593 | 1 |
|  | REV: 5′-TGGGTRAARTARGTSACCAGA-3′ |  |  |  |
| *bla*VEB-type | FW: 5’- CGACTTCCATTTCCCGATGC-3’ | 54 | 642 | 2 |
|  | REV: 5’- GGACTCTGCAACAAATACGC-3’ |  |  |  |
| *bla*KPC-type | FW: 5′-TGTCACTGTATCGCCGTC-3′ | 55 | 1,000 | 3 |
|  | REV: 5′-CTCAGTGCTCTACAGAAAACC-3′ |  |  |  |
| *bla*OXA-48-type | FW: 5′-TTGGTGGCATCGATTATCGG-3′ | 52 | 743 | 4 |
|  | REV: 5′-GAGCACTTCTTTTGTG ATGGC-3′ |  |  |  |
| *aac(6')-Ib-cr* | FW: 5'-TTGCGATGCTCTATGAGTGGCTA-3' | 55 | 482 | 5 |
|  | REV: 5'-CTCGAATGCCTGGCGTGTTT-3' |  |  |  |
| *armA* | FW: 5'-ATTCTGCCTATCCTAATTGG-3' | 55 | 315 | 6 |
|  | REV: 5'-ACCTATACTTTATCGTCGTC-3' |  |  |  |
| *aphA6* | FW: 5'-ATGGAATTGCCCAATATTATTC-3' | 49 | 780 | 7 |
|  | REV: 5'-TCAATTCAATTCATCAAGTTTTA-3' |  |  |  |
| *bla*DHA-type | FW: 5'-AACTTTCACAGGTGTGCTGGGT-3' | 50 | 405 | 8 |
|  | REV: 5'-CCGTACGCATACTGGCTTTGC-3' |  |  |  |
| *bla*CMY-type | Fw: 5’- GATGGCAARGCCCACTAYTTC-3’ | 46 | 916 | 9 |
|  | Rev: 5’-TTGGCCAGCATGACGATG-3’ |  |  |  |
| *bla*MOX-type | Fw: 5’-GATGGCAARGCCCACTAYTTC-3’ | 46 | 916 | 9 |
|  | Rev: 5’-TTGGCCAGCATGACGATG-3’ |  |  |  |
| *qnrB* | Fw: 5′-GATCGTGAAAGCCAGAAAGG-3’ | 55 | 469 | 10 |
|  | Rev: 5′-ACGATGCCTGGTAGTTGTCC-3’ |  |  |  |
| *qnrS* | Fw: 5′-ACGACATTCGTCAACTGCAA-3’ | 55 | 417 | 10 |
|  | Rev: 5′-TAAATTGGCACCCTGTAGGC |  |  |  |

**Supplementary Table 2. Chemical composition of the surface and ground waters sampled**

| **Sample** | **Water typology** | **t**  **(°C)** | **E.C.***  **μS/cm** | **pH** | **Na^+^**  **mg/L** | **K^+^**  **mg/L** | **Mg^2+^**  **mg/L** | **Ca^2+^**  **mg/L** | **Cl^-^**  **mg/L** | **NO_3_^-^**  **mg/L** | **SO_4_^2-^**  **mg/L** | **HCO_3_^-^**  **mg/L** | **COD^a^**  **mg/L** |
| --- | --- | --- | --- | --- | --- | --- | --- | --- | --- | --- | --- | --- | --- |
| 1 | Stream | 14.5 | 276 | //^b^ | // | // | // | // | // | // | // | // | 31,66 |
| 2 | Small lake | 17.4 | 458 | 7.5 | 9.9 | 1.6 | 10.8 | 57.5 | 19.2 | 6.4 | 40.5 | 145 | 43.1 |
| 4 | Spring | 14.6 | 469 | 7.4 | 24.3 | 2.7 | 7.6 | 49.0 | 31.0 | 45.5 | 24.6 | 114 | 36.6 |
| 5 | Pond | 16.7 | 518 | 7.6 | 23.8 | 3.6 | 8.5 | 44.0 | 45.9 | 3.2 | 36.5 | 107 | 56.8 |
| 6 | Stream | 15.0 | 294 | 7.5 | 9.0 | 2.1 | 6.3 | 37.4 | 15.6 | 7.2 | 45.2 | 84 | 33.1 |
| 7 | Canal | 14.7 | 277 | 7.4 | 7.7 | 1.9 | 5.9 | 35.0 | 14.1 | 6.6 | 44.6 | 76 | 28.9 |
| 8 | Spring | 13.4 | 516 | 7.5 | 15.2 | 1.0 | 11.9 | 61.5 | 46.2 | 3.0 | 37.5 | 153 | 37.4 |
| 9 | Sewage wastewater | 15.8 | 649 | 7.6 | 31.5 | 2.1 | 12.8 | 70.6 | 51.2 | 6.5 | 58.5 | 175 | 53.9 |
| 10 | Canal | 17.8 | 276 | 7.7 | 9.0 | 1.9 | 6.4 | 37.0 | 13.8 | 5.2 | 42.5 | 84 | 30.6 |
| 11 | Pond | 14.6 | 577 | 7.4 | 10.3 | 1.4 | 14.3 | 72.3 | 49.3 | 10.1 | 39.2 | 168 | 25.1 |
| 12 | Spring | 15.7 | 270 | 7.5 | 13.9 | 1.5 | 8.5 | 50.6 | 30.9 | 60.6 | 37.6 | 53 | 26.2 |
| 13 | Treatment plants | 17.8 | 478 | 7.6 | 36.3 | 10.0 | 7.1 | 36.0 | 36.5 | 18.9 | 41.6 | 107 | 35.0 |
| 15 | Canal | 14.5 | 188 | 7.7 | 6.2 | 1.8 | 3.5 | 22.4 | 10.7 | 2.5 | 29.5 | 46 | 28.0 |
| 16 | Canal | 14.4 | 184 | 7.5 | 4.2 | 1.3 | 3.3 | 24.5 | 11.0 | 2.7 | 30.3 | 46 | 30.0 |
| 17 | Canal | 15.0 | 171 | 7.6 | 5.6 | 1.7 | 4.6 | 27.3 | 11.0 | 2.1 | 41.8 | 61 | 29.8 |
| 18 | Canal | 15.2 | 185 | 7.8 | 5.5 | 1.8 | 4.9 | 28.2 | 12.2 | 2.5 | 40.9 | 61 | 30.1 |
| 21 | Canal | 14.9 | 248 | 7.4 | 7.5 | 2.6 | 4.8 | 30.2 | 13.1 | 2.9 | 43.9 | 69 | 29.7 |
| 22 | Stream | 15.0 | 319 | // | // | // | // | // | // | // | // | // | 30.8 |

# *E.C.: electric conducibility; ^a^COD: Chemicals Oxygen Demand; ^b^//: not tested.

**Supplementary Table 3. Bacterial counts on Plate Count Agar plates, MacConkey Agar plates, and MacConkey Agar plates supplemented with different concentrations of cefotaxime and meropenem**

| **Site** | **PCA^a^**  **CFU ml^-1^** | **MC^b^**  **CFU ml^-1^** | **MC + 1μg/ml CTX^c^**  **CFU ml^-1^** | **MC + 2μg/ml CTX**  **CFU ml^-1^** | **MC + 0.25μg/ml MEM^d^**  **CFU ml^-1^** | **MC+ 4μg/ml MEM**  **CFU ml^-1^** |
| --- | --- | --- | --- | --- | --- | --- |
| **1** | 750 | 300 | 115 | 28 | 17 | 0 |
| **2** | 108 | 20 | 20 | 9 | 5 | 0 |
| **4** | 320 | 74 | 74 | 16 | 3 | 0 |
| **5** | 240 | 26 | 26 | 18 | 13 | 0 |
| **6** | 450 | 250 | 34 | 15 | 6 | 0 |
| **7** | 380 | 215 | 18 | 13 | 16 | 0 |
| **8** | 440 | 200 | 3 | 1 | 3 | 0 |
| **9** | 2400 | 2500 | 185 | 131 | 32 | 0 |
| **10** | 740 | 710 | 73 | 51 | 33 | 0 |
| **11** | 61 | 35 | 5 | 5 | 0 | 0 |
| **12** | 730 | 470 | 213 | 144 | 23 | 1 |
| **13** | 100 | 61 | 42 | 14 | 7 | 0 |
| **15** | 115 | 41 | 13 | 1 | 0 | 0 |
| **16** | 51 | 13 | 10 | 5 | 1 | 0 |
| **17** | 300 | 150 | 40 | 26 | 32 | 0 |
| **18** | 445 | 355 | 94 | 69 | 56 | 7 |
| **21** | 570 | 290 | 90 | 65 | 5 | 0 |
| **22** | 565 | 315 | 16 | 10 | 1 | 0 |

^a^PCA: Plate Count Agar

^b^MC: MacConkey Agar

^c^CTX: Cefotaxime

^d^MEM: Meropenem

**Supplementary Table 4.** Co-resistance of genes identified in E. coli and K. pneumoniae isolates

| **Isolate** | ***bla*CTX-M-type** | ***bla*VEB-type** | ***bla*MOX-type** | ***bla*OXA-48*-*type** | ***bla*KPC*-*type** | ***aac(6')-Ib-cr*** | ***qnrS*** |
| --- | --- | --- | --- | --- | --- | --- | --- |
| **C1 *E. coli*** | + |  |  |  |  | + |  |
| **C1 *K. pneumoniae*** | + |  |  |  | + | + |  |
| **C2 *E.coli*** | + |  |  |  |  | + |  |
| **C4 *K. intermedia*** |  |  |  |  |  |  |  |
| **C6 *E. coli*** | + |  |  |  |  | + |  |
| **C6 *C. freundii*** |  |  |  |  |  |  |  |
| **C7-2 *E. coli*** | + |  |  |  |  |  |  |
| **C7 *E. coli*** | + |  |  | + |  |  |  |
| **C9 *K. oxytoca*** |  |  |  |  |  |  |  |
| **C9-1 *E. coli*** | + |  |  |  |  |  |  |
| **C9-2 *E. coli*** | + |  |  | + |  |  |  |
| **C9-3 *E. coli*** | + |  |  | + |  |  |  |
| **C9-4 *E. coli*** | + |  |  | + |  |  |  |
| **C10-2 *E. coli*** | + |  |  |  |  | + |  |
| **C10-1 *E. coli*** | + |  |  |  |  | + |  |
| **C10-3 *E. coli*** | + |  |  |  |  | + |  |
| **C11-1 *E. coli*** | + |  |  |  |  |  |  |
| **C11-2 *E. coli*** | + |  |  |  |  |  |  |
| **C12 *K*. *intermedia*** |  |  |  |  |  |  |  |
| **C13 *C. freundii*** |  |  |  |  |  |  |  |
| **C17 *K. pneumoniae*** | + |  |  |  |  |  | + |
| **C17 *E. coli*** | + |  |  |  |  |  | + |
| **C17-2 *K. pneumoniae*** | + |  |  |  |  |  | + |
| **C21-4 *E. coli*** | + |  |  |  |  |  |  |
| **C21-2 *E. coli*** | + |  |  |  |  | + |  |
| **C21-3 *E. coli*** | + |  |  |  |  | + |  |
| **C21 *E. coli*** | + |  |  |  |  |  |  |
| **C22-2 *E. coli*** |  | + |  |  |  |  |  |
| **C22 *K. aerogenes*** |  |  |  |  |  |  |  |
| **C22 *E. coli*** | + |  |  |  |  |  | + |

**Supplementary Table 5. Susceptibility profiles and WGS results for C7 and C9-3 *E. coli* isolates**

| Antibiotic class | Antibiotic name | C7 *E. coli* | | | C9-3 *E. coli* | | |
| --- | --- | --- | --- | --- | --- | --- | --- |
|  |  | MIC^a,b,c^ | Resistance determinants | | MIC | Resistance determinants | |
|  |  |  | Chromosomal | Plasmid |  | Chromosomal | Plasmid |
| β-lactams | Amoxicillin | >8/4 R | *bla*CTX-M-15  *bla*OXA-244 |  | >8/4 R | *bla*CTX-M-15  *bla*OXA-244 |  |
|  | Ampicillin | >8 R |  |  | >8 R |  |  |
|  | Piperacillin | >16 R |  |  | >16 R |  |  |
|  | Aztreonam | >4 R |  |  | >4 R |  |  |
|  | Cefuroxime | >8 R |  |  | >8 R |  |  |
|  | Cefotaxime | >16 R |  |  | >16 R |  |  |
|  | Ceftazidime | >8 R |  |  | >8 R |  |  |
|  | Cefepime | >8 R |  |  | >8 R |  |  |
|  | Piperacillin/Tazobactam | >16 R |  |  | >16 R |  |  |
|  | Ertapenem | <=0.5 S |  |  | <=0.5 S |  |  |
|  | Meropenem | 2 S |  |  | 2 S |  |  |
|  | Imipenem | <=1 S |  |  | <=1 S |  |  |
| Fluoroquinolones | Ciprofloxacin | >1 R | *parE* mutation (p.I529L)  *parC* mutation (p.S80I)  *parC* mutation (p.E84V)  *gyrA* mutation (p.S83L) |  | >1 R | *parE* mutation (p.I529L)  *parC* mutation (p.S80I)  *parC* mutation (p.E84V)  *gyrA* mutation (p.S83L)  *mdf(A)* |  |
|  | Levofloxacin | 1 R |  |  | 1 R |  |  |
| Aminoglycosides | Gentamicin | <=2 S |  | *aadA5* | <=2 S |  | *aadA5* |
|  | Amikacin | <=8 S |  |  | <=8 S |  |  |
|  | Tobramycin | <=2 S |  |  | <=2 S |  |  |
| Tetracyclines | Tetracycline | <=8 S | *mdf(A)* |  | <=8 R | *mdf(A)* |  |
|  | Tigecycline | <=1 S |  |  | <=1 S |  |  |
| Phenicols | Chloramphenicol | <=8 S | *mdf(A)* |  | >8 R | *mdf(A)* |  |
| Polymixins | Colistin | <=2 S |  |  | <=2 S |  |  |
|  | Fosfomycin | <=32 S |  |  | <=32 S |  |  |
| Sulfonamides | Trimethoprim/Sulfamethoxazole | <=2/38 S |  | *dfrA17*  *sul1* | >4/76 R |  | *dfrA17*  *sul1* |
| Macrolides | Erythromycin | NT | *mdf(A)* | *erm(B)* | NT | *mdf(A)* | *erm(B)* |

^a^R: Resistant; ^b^S: Susceptible; ^c^NT: Not tested.

**References**

1. Pagani L, Dell'Amico E, Migliavacca R, D'Andrea MM, Giacobone E, Amicosante G, Romero E, Rossolini GM. Multiple CTX-M-type extended-spectrum beta-lactamases in nosocomial isolates of *Enterobacteriaceae* from a hospital in northern Italy. J Clin Microbiol. 2003 Sep;41(9):4264-9. doi: 10.1128/JCM.41.9.4264-4269.2003.
2. Lee S, Park YJ, Kim M, Lee HK, Han K, Kang CS, Kang MW. Prevalence of Ambler class A and D beta-lactamases among clinical isolates of *Pseudomonas aeruginosa* in Korea. J Antimicrob Chemother. 2005 Jul;56(1):122-7. doi: 10.1093/jac/dki160.
3. Yigit H, Queenan AM, Anderson GJ, Domenech-Sanchez A, Biddle JW, Steward CD, Alberti S, Bush K, Tenover FC. Novel carbapenem-hydrolyzing beta-lactamase, KPC-1, from a carbapenem-resistant strain of *Klebsiella pneumoniae*. Antimicrob Agents Chemother. 2001 Apr;45(4):1151-61. doi: 10.1128/AAC.45.4.1151-1161.2001.
4. Poirel L, Héritier C, Tolün V, Nordmann P. Emergence of oxacillinase-mediated resistance to imipenem in *Klebsiella pneumoniae*. Antimicrob Agents Chemother. 2004 Jan;48(1):15-22. doi: 10.1128/AAC.48.1.15-22.2004.
5. Park CH, Robicsek A, Jacoby GA, Sahm D, Hooper DC. Prevalence in the United States of *aac(6')-Ib-cr* encoding a ciprofloxacin-modifying enzyme. Antimicrob Agents Chemother. 2006 Nov;50(11):3953-5. doi: 10.1128/AAC.00915-06.
6. Doi Y, Arakawa Y. 16S ribosomal RNA methylation: emerging resistance mechanism against aminoglycosides. Clin Infect Dis. 2007 Jul 1;45(1):88-94. doi: 10.1086/518605.
7. Hujer KM, Hujer AM, Hulten EA, Bajaksouzian S, Adams JM, Donskey CJ, Ecker DJ, Massire C, Eshoo MW, Sampath R, Thomson JM, Rather PN, Craft DW, Fishbain JT, Ewell AJ, Jacobs MR, Paterson DL, Bonomo RA. Analysis of antibiotic resistance genes in multidrug-resistant *Acinetobacter* spp. isolates from military and civilian patients treated at the Walter Reed Army Medical Center. Antimicrob Agents Chemother. 2006 Dec;50(12):4114-23. doi: 10.1128/AAC.00778-06.
8. Pérez-Pérez FJ, Hanson ND. Detection of plasmid-mediated AmpC beta-lactamase genes in clinical isolates by using multiplex PCR. J Clin Microbiol. 2002 Jun;40(6):2153-62. doi: 10.1128/JCM.40.6.2153-2162.2002.
9. D'Andrea MM, Nucleo E, Luzzaro F, Giani T, Migliavacca R, Vailati F, Kroumova V, Pagani L, Rossolini GM. CMY-16, a novel acquired AmpC-type beta-lactamase of the CMY/LAT lineage in multifocal monophyletic isolates of *Proteus mirabilis* from northern Italy. Antimicrob Agents Chemother. 2006 Feb;50(2):618-24. doi: 10.1128/AAC.50.2.618-624.2006.
10. Robicsek A, Strahilevitz J, Sahm DF, Jacoby GA, Hooper DC. qnr prevalence in ceftazidime-resistant *Enterobacteriaceae* isolates from the United States. Antimicrob Agents Chemother. 2006 Aug;50(8):2872-4. doi: 10.1128/AAC.01647-05.
